# Trends in botanical exploration in Nigeria forecast over 1000 yet undescribed vascular plant species

**DOI:** 10.1093/aob/mcad106

**Published:** 2024-05-10

**Authors:** Abubakar Bello, Stewart M Edie, Kowiyou Yessoufou, Alexandra Nora Muellner-Riehl

**Affiliations:** Department of Biology, Umaru Yar’adua University, P.M.B. 2218, Katsina, Nigeria; Department of Molecular Evolution and Plant Systematics & Herbarium (LZ), Institute of Biology, Leipzig University, Johannisallee 21–23, D-04103 Leipzig, Germany; German Centre for Integrative Biodiversity Research (iDiv) Halle-Jena-Leipzig, Puschstraße 4, D-04103 Leipzig, Germany; Department of Paleobiology, National Museum of Natural History, Smithsonian Institution, Washington, DC, USA; Department of Geography, Environmental Management and Energy Studies, University of Johannesburg, Auckland Park 2006, Johannesburg, South Africa; Department of Molecular Evolution and Plant Systematics & Herbarium (LZ), Institute of Biology, Leipzig University, Johannisallee 21–23, D-04103 Leipzig, Germany; German Centre for Integrative Biodiversity Research (iDiv) Halle-Jena-Leipzig, Puschstraße 4, D-04103 Leipzig, Germany

**Keywords:** Biodiversity, conservation, Nigeria, species description, taxonomic expertise, taxonomic impediment, vascular plants

## Abstract

**Background and aims:**

Taxonomists are primary actors of biodiversity assessment. At the same time, there is awareness by the taxonomic community at large that the field is going through a crisis, sometimes referred to as the ‘taxonomic impediment’. Coupled with the ongoing biodiversity crisis, or 6th mass extinction, this biodiversity impedance puts at risk the target set in the Convention on Biological Diversity’s (CBD) Global Biodiversity Framework vision 2050, which calls for urgent action to ‘… put biodiversity on a path to recovery by 2030 for the benefit of planet and people’. This risk is particularly pronounced in tropical African countries where taxonomic studies are done on an *ad hoc* basis. In this study, our aim is to investigate the historical trends in botanical exploration of vascular plants in Nigeria and forecast the near-term (50-year) description of presently unknown species, which we use to discuss scenarios of taxonomic effort that may be necessary for a comprehensive biodiversity assessment in the country.

**Methods:**

The study is based on a dataset from the World Checklist of Vascular Plants, containing all vascular plant species reported to occur in Nigeria. We fit nested Bayesian time series regressions to estimate the long-term trend in the rate of description of vascular plant species in Nigeria. From these models, we use an ensemble forecast to estimate the number of species descriptions by the year 2070, and then evaluate the description rates per taxonomist required to meet this estimate under different totals of active taxonomists.

**Key results:**

We find a striking difference in species description between Nigerian botanists and their foreign counterparts, with the former contributing relatively small numbers. Additionally, only a fraction of the authors involved in describing Nigeria’s vascular plants are of indigenous origin. Our study reveals that the number of new species described annually exhibits a long-term increasing trend, with an average of 19.5 species described per year. However, after taking into account year-to-year variability and the number of taxonomists active in a given year, the long-term trend in species descriptions credibly declines over time. While the number of authors involved in describing species has generally increased over time, it has remained stable since the 1950s. Predictions for the number of new species descriptions by 2070 vary by model, with an ensemble prediction estimating 1140 species descriptions, but ranging from 1004 to 2239 between individual models.

**Conclusions:**

The study estimates that current levels of taxonomic activity should lead to a 20 % increase in known species of vascular plants in Nigeria over the next 50 years, which is still probably an underestimate of the true, unknown species richness. Urgent action is needed to address the taxonomic impediment so that local taxonomic studies in tropical African countries can achieve the CBD’s Global Biodiversity Framework vision 2050. Here, we outline some key pathways to achieving this goal.

## INTRODUCTION

The ongoing biodiversity crisis and taxonomic impediment pose a threat to the target set in the Convention on Biological Diversity (CBD) Global Biodiversity Framework (GBF) vision 2050. The GBF calls for urgent action to recover biodiversity by 2030 for the benefit of both the planet and people, and a key part of the Kunming–Montreal GBF is the goal of a 10-fold increase of the rate of species description by 2050 (Convention on Biological Diversity, 2022). Similarly, many international organizations, including the International Union for the Conservation of Nature (IUCN), have called for a hastening of species description to better understand and protect biodiversity. The GBF also includes provisions for funding biodiversity-related projects, such as public and private grants for species description and habitat restoration (Convention on Biological Diversity, 2022). However, the shortage of taxonomists, especially in tropical African countries where taxonomic studies are sporadic, is hindering the achievement of these goals. In addition to the low number of taxonomists, publication practices in taxonomy are often neglected as a taxonomic impediment (Grieneisen *et al*., 2014; Hobern *et al*., 2021; Lien *et al*., 2021; Thomson *et al*., 2021). A worldwide survey with taxonomists revealed that over half of the respondents are established/late-career researchers, while only a relatively low number of early-career researchers and graduate students are actively involved in taxonomic practice, pointing towards an ageing taxonomist population (Agnarsson and Kuntner, 2007; Salvado *et al*., 2022). Therefore, addressing both taxonomist shortages and publication practices in taxonomy is crucial for achieving the GBF vision 2050 and mitigating the ongoing biodiversity crisis.

In Nigeria, many of the described and reported plant species are based on the work of non-Nigerian taxonomists (Hutchinson, 1921*a*, *b*; Hutchinson and Dalziel, 1927; Keay, 1954; Hepper, 1963). However, to date, many of these species have been insufficiently investigated and/or taxonomically revised, and taken together with the scarcity of indigenous taxonomic expertise, sufficient information or updates are not available (Klopper *et al*., 2002). Moreover, Nigeria and many other countries with developing economies lack biodiversity databases and fail to recognize the importance of biological diversity in the national economy, which raises serious concerns about their ability to fulfil the endorsed obligations to the CBD (Federal Ministry of Environment, 2001; National Biodiversity Strategy and Action Plan, 2006). Documenting the full account of plant diversity is essential for protecting threatened species and ensuring they can sustain human needs before they become extinct (Convention on Biological Diversity, 2010). This study is part of a planned series of publications funded by the German Centre of Integrative Biodiversity Research (iDiv) to update species diversity data from Nigeria, which will help Nigeria fulfil its national obligations to all signed international treaties and agreements and support plant species diversity-related indicators in the CBD’s post-2020 GBF.

**Fig. 1.**
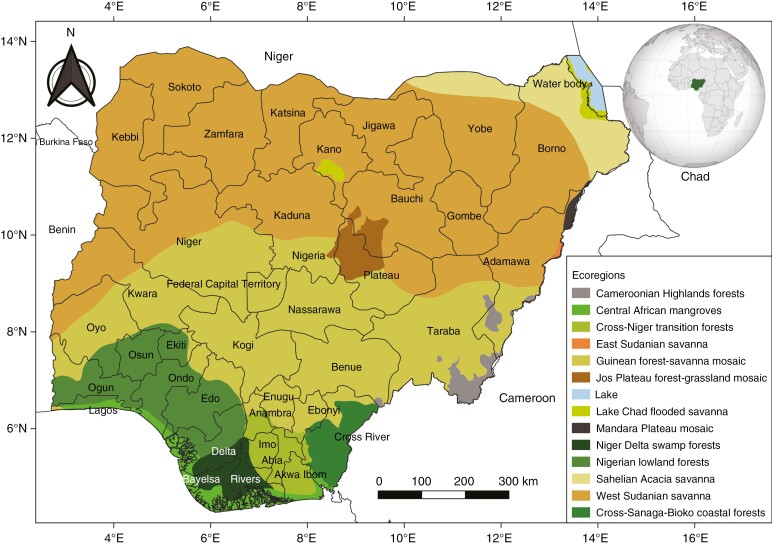
Map of Nigeria showing ecoregions. Classification follows WWF ecoregions of the world.

Nigeria is located between longitudes 3°E and 15°E and latitudes 4°N and 14°N. It comprises 36 states, 774 local governments and over 250 ethnic groups, and has a population of over 210 million in a land area of 923 768 km^2^ (National Biodiversity Strategy and Action Plan, 2006; David, 2008). Nigeria has diverse landscapes and ecosystems, ranging from forests in the south to moist savannas in the central part of the country and dry arid savannas in the extreme north (see Fig. 1). As a signatory to the CBD and other international treaties on biodiversity and conservation, Nigeria is obligated to document its plant diversity. However, except for a few Flora treatments of restricted plant families, such as Flora of Nigeria-Grasses (Poaceae; Keay, 1991) and Flora of Nigeria-Sedges (Cyperaceae; Lowe, 1974), there is currently no comprehensive Flora of Nigeria. Despite significant investment, several initiatives to develop the Flora of Nigeria failed, resulting in frequently erratic, contradictory or grossly misleading estimates of plant diversity due to the lack of a comprehensive checklist or Flora. These initiatives include the establishment of the Botanical Society of Nigeria (BOSON) and the Nigerian Society for Conservation Biology (NSCB). A project by BOSON to create a comprehensive inventory of Nigerian plant species was abandoned due to lack of funding and resources. To address this deficiency, an appropriate synthesis of the immense plant diversity and information in Nigeria is necessary as the first step toward the creation of an exhaustive Flora of Nigeria. This data-driven process will improve the quality and authenticity of plant diversity records from Nigeria and facilitate the creation of suitable conservation strategies and assessments to reduce the loss of plant diversity.

According to the latest data available, there an estimated 6000 vascular plant species have been documented for Nigeria, of which ~5627 are native [National Biodiversity Strategy and Action Plan, 2006; Plant of the World Online (POWO, 2021)]. This study seeks to answer the following questions: (1) Who were the key contributors to botanical exploration in Nigeria? (2) What is the overall temporal pattern of botanical species description in Nigeria? (3) Based on the historical trend of species description in Nigeria, how many vascular plant species could be described in the next 50 years? We then discuss how potential changes to future taxonomic effort could impact the goal of providing a comprehensive biodiversity assessment in the country, followed by approaches to mitigating impacts of the ongoing and impending taxonomic impediment.

## MATERIALS AND METHODS

### Data compilation

The data used in this study were obtained from the World Checklist of Vascular Plants (WCVP; Govaerts *et al*., 2021; Supplementary Data Table S1) provided by the Royal Botanic Gardens, Kew, United Kingdom (RBGK). However, it is important to note that the WCVP and its associated World Checklist of Selected Plant Families (WCSP) are no longer available online. These resources have been transitioned to a new platform, ‘Plants of the World Online’ (POWO: https://powo.science.kew.org/). Only currently accepted species are analysed here. Both the number of species described and the number of unique authors describing those species were tallied per year. The time series begins in 1753 and ends in 2020. Years in which no species were described were set to zero counts for species and 0.1 counts for number of unique authors (positive values were needed to model this term as a logged offset, see below).

### Modelling trends in species description

**Fig. 2.**
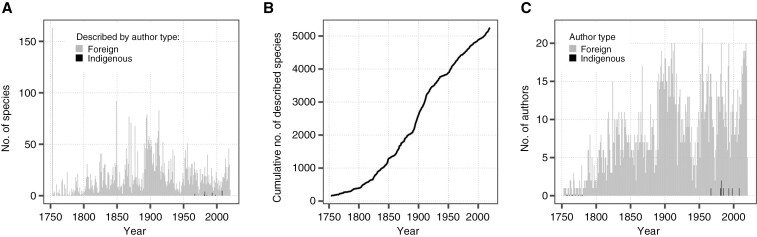
Species description of Nigerian vascular plants from 1753 to 2020. (A) Counts of species described per year. (B) Cumulative counts of species described through time. (C) Counts of authors describing species in a given year.

The count of species described per year was modelled using a series of nested Bayesian linear regressions (Table 1; Edie *et al*., 2017). The null, model 1, estimates the average count of species described per year over the study interval. Counts were then allowed to vary linearly with time (model 2), and by assuming an added 1-year autoregressive term (model 3). The 1-year autoregressive term takes into account the potential year-to-year dependence of species descriptions, perhaps better capturing the episodic bursts of sampling through time seen as secular fluctuations in description counts in Fig. 2A. Finally, counts were modelled as a function of time, adding the natural log of the number of unique authors in a given year as an offset term (i.e. an exposure; model 4), and then including the 1-year autoregressive term (model 4.1). Including the number of unique authors as an exposure variable, or offset term, allows the effect of time on counts of species descriptions to be evaluated against the potentially increased opportunity for discovery with increasing number of authors (see Winter and Bürkner, 2021 for this general approach); this assumes more authors reflect greater opportunity for species discovery, but may also reflect, in part, trends in scientific culture, where more authors are now being included in species descriptions. Including the offset term transforms the interpretation of the long-term trend to the yearly rate of species description per author.

**Table 1.** Nested model of species description. Terms: *count* is the number of species described in a given calendar *year*; *author* is the number of unique authors named on a species description in a given year. Model support was assessed by leave-one-out cross-validation and is shown as the differences in expected log predictive density ‘Δelpd’ with the standard error (s.e.). The best supported model has an elpd of zero.

Model	*brms* formula	Description	Δelpd (s.e.)
Model 1	*count* ~ 1	Average count of species described per year	−199.9 (11.5)
Model 2	*count* ~ *year*	Count of species described per year varies linearly with time	−196.4 (12.1)
Model 3	*count* ~ *year + *ar(p = 1)	As in model 2, adding a 1-year autoregressive term.	−119.1 (9.7)
Model 4	*count* ~ *year + *offset(log(*author*))	As in model 2, adding an offset as the logged number of unique authors describing species in a given year	−136.0 (50.4)
Model 4.1	*count* ~ *year + *offset(log(*author*))* + *ar(p = 1)	As in model 4, adding a 1-year autoregressive term	0 (0) [best supported model]

All models were fit using the R package *brms* (Bürkner, 2017) assuming a negative binomial response distribution with a log-link given that the spread of per-year count values ranges from 0 to 163; not shown here are the underestimates of count values when assuming Poisson response distributions and that both zero-inflated Poisson and zero-inflated negative binomial response distributions overpredicted the frequency of years with low count values. Default priors specified by *brms* are used per model type (details in Supplementary Data Tables S2–S6). All models were fit using four chains over 7500 iterations (15 000 for models 3, 4 and 4.1), sampled every ten chains for a total of 2600 post-warmup posterior samples (5200 for models 3, 4 and 4.1). Details on chain mixing and model convergence are provided in Tables S2–S6. Model performance was evaluated using leave-one-out cross-validation (Vehtari *et al*., 2017).

### Forecasting species description

Species descriptions were forecast through the year 2070 by simulating trends forward in time from the posterior distributions of each fitted model (Hyndman and Athanasopoulos, 2018). Forecasts for models 4 and 4.1 required estimates on the future number of authors per year; these counts were randomly sampled from the observed values between 2000 and 2020, which we assume to best represent the expected taxonomic effort into the near future. An ensemble forecast was made by averaging predictions over all model posteriors, weighted by their expected log predictive density as determined via the leave-one-out-cross-validation.

## RESULTS

### History of botanical explorations in Nigeria

Botanical exploration in Nigeria began with European explorers in the 15th century who visited the West African coast (Holland, 1908). However, serious botanical exploration did not start until the 19th century. European botanists began exploring the Niger Delta region in the early 19th century. Notable explorers included William Jameson Hooker (Hooker, 1872), Thomas Edward Bowdich (Bowdich, 1819) and Friedrich Gerhard Rohlfs (Rohlfs, 1884). They collected plant specimens that were sent to herbaria in Europe (Holland, 1908). These expeditions added to the knowledge of the West African flora. More intensive botanical exploration occurred in the early 20th century during British colonial rule. Explorers included Percy W. Richards (Richards 1939), E. W. S. Craig (Craig, 1927) and J. M. Dalziel (Dalziel, 1937). They explored regions across Nigeria, collected thousands of plant specimens (Hutchinson and Dalziel, 1954–1972; Hepper, 1967, 1972) and produced many publications on Nigerian plants (Dalziel, 1937; Unwin, 1937; Keay, 1954; Cooper, 1957; Ghazanfar, 1989). Forestry Herbarium (FHI) at the Forestry Research Institute of Nigeria was also established at the time in 1954. After Nigeria’s independence in 1960, botanical research and exploration continued at Nigerian universities and research institutes. Notable botanists include Professors Onochie, Charles Francis Akado (1914–); Okafor, Jonathan Chukuemeka (1934-–); Soladoye, Michael O. (1945–); Moses A. Isawumi (1995–); and Daramola, Benjamin Obajide (collector; 1933–). They have described new plant species, studied plant diversity and contributed to the conservation of plant genetic resources in Nigeria (Onochie, 1968; Akobundu and Agyakwa, 1998; Akoègninou *et al*., 2006).

The first significant work in connection with tropical Africa was The Niger Flora, published in 1819 by Sir W. J. Hooker and Mr George Bentham (Holland, 1908). It was based on vascular plant collections made by Dr Theodore Vogel and Mr Ansell during the Niger Expedition of 1811 (Holland, 1908). The Flora of Tropical Africa was prepared at the RBGK and was sanctioned by the Treasury in 1864. Professor D. Oliver edited three volumes between 1868 and 1877 (Oliver, 1868), and in 1891, its preparation was resumed under the editorship of Sir W. T. Thiselton-Dyer. Nine volumes have been published in total (Holland, 1908). The Flora of Tropical Africa has contributed significantly to the vascular plant botanical survey of tropical Africa. Volume III contains 1134 vascular plant species, and volume IV had 2176 species for the entire flora of Tropical Africa, double the original estimate in only 11 years, leading to its division into two parts. The increase in species recovered for Tropical Africa was very unequal in different plant orders, with the largest orders experiencing an over 300 % increase. The significant increase in knowledge of the flora of Tropical Africa since 1891 has been due to old collections being systematically studied, fresh collections being made in additional countries and botanical stations, and Germany’s energy in the botanical survey of its colonies. Table 2 provides the names of collectors who have supplied specimens from Nigeria to the RBGK or the Natural History Museum in London. However, the list covers the beginning of the 19th century to the beginning of the 20th century only.

**Table 2.** Names of collectors who have supplied specimens from Nigeria to the Royal Botanic Gardens, Kew, or the British Museum, in chronological order.

Name	Years active	Location	Genera/species named after them
Mungo Park, A.L.S	1795–7, 1801–5	Africa	*Parkia* R.Br.
Captain Hugh Clapperton	1822–4	Sokoto province; buried at Jungeri, was the first European to visit Sokoto, 16 March 1821	*Clappertonia*. Meisn. = *Honckenya* Willd.
Lieut.-Col. Dixon Denham	1822–4	Sierra Leone; died, Sierra Leone, 8 May 1828	*Denhamia* Schoti = *Culcasia* R.Br.
Walter Oudney	1821–4	Bornu and W. Sudan, died at Murmur, W. Sudan, 12 January 1884	*Oudneya* R.Br.
Dr Theodore Vogel	1841	Near Lokoja	–
John Ansell	1841	Niger Expedition	*Ansellia* Lindl.
Edward Vogel	1851–5	Bornu	–
Edward George Irving, M.D., Surgeon, R.N.	1844–55	Abeokuta, etc.	*Irvingia* Hook.f.
Miss Gurney	1855	Lagos	–
Dr William Balfour Baikie	1855–64	Nupe, etc.	*Baikiaea* Hook.f.
Charles Barter	1857–9	Rabba, Nupe and Borgu; died at Rabba, July 1859	*Barteria* Hook.f.
Gustav Mann	1860	Old Calabar, Bonny, etc.	–
Rev. William C. Thomson	1863	Old Calabar	–
William Grant Milne	1862–6	West Coast of Africa; died at Creektown, Old Calabar, 3 May 1866	–
W. Kalbreyer	1877–84	Niger Delta	–
Rev. Hugh Goldie	1888	Old Calabar district	*Aristolochia* g*oldieana* Hook.f.
Sir H. H. Johnston, G.C.M.G.	1888	Cross River	–
Walter Higginson	1890	Lagos	–
Alvan Millson	1890–1	Lagos and Yoruba	–
Dr John William Rowland	1890–3	Lagos	–
Sir George Chardin Denton, K.C.M.G.	1895	Deputy Governor of Lagos	–
Sir Alfred Moloney, K.C.M.G.	1882–96	Lagos	–
Henry Millen	1892–6	Curator of the Botanic Garden, Lagos	–
Horace Walter Leigh ton Billington	1893–7	First Curator of Botanic Gardens, Old Calabar	–
John Henry Holland	1897–1900	Curator, Botanic Gardens, Old Calabar	–
Captain Arthur Johnstone Richardson	1898	Near Lokoja	–
Harold Buchan Lloyd	1898	Assistant Curator, Botanic Gardens, Old Calabar	–
Dr Ernest Ule	1899	Old Calabar	–
T. B. Dawodu	1899	Lagos	–
Cyril Punch	1900	Lagos (First Superintendent of Forests)	–
L. Kentish Rankin	1901	Northern Nigeria	–
Sir William MacGregor	1901	Lagos	–
W. R. Elliott	1903	Various parts from the Lagos frontier to Lake Chad; died 13 March 1908, at Bedford whilst on leave	–
P. H. Talbot	1904	From lbi on the Benue to Lake Chad, through Bauchi	–
Captain G. B. Gosling	1904	Benue to Lake Chad	–
Dr J. M. Dalziel	1905	Lokoja, Zungeru, Kontagora, etc.	–
Norman C. MacLeod	1905	Deputy Conservator of Forests; Southern Nigeria	–
H. N. Thompson	1906	Conservator of Forests; Southern Nigeria	–
Dr A. H. Unwin	1906	Assistant Conservator of Forests; Southern Nigeria	–
E. W. Foster	1906	Lagos, Assistant Conservator of Forests	–
J. C. Leslie	1906	Assistant Conservator of Forests, Asaba; Southern Nigeria	–
Col. E. J. Lugard, D.S.O.	1907	Zungeru and Lokoja, Northern Nigeria	–
G. C. Dudgeon	1907	Superintendent of Agriculture; West Coast of Africa	–
R. E. Dennet	1907	Assistant Conservator of Forests; Southern Nigeria	–
H. Dodd	1908	Curator, Botanical Department; Southern Nigeria	–
R.W.J. Keay	1936–63	South and Northern Nigeria	*Keayodendron* Leandri

### Species description through time

Qualitatively, after an initial pulse of 163 plant species native to Nigeria described in 1753 by Linneaus, species description accelerated until a slightly decreased rate in the 1890s, but then increased at its greatest rate between ~1890 and 1910 before settling into a lower rate of description of 20–30 species per year over the past century (Fig. 2A, B). The number of authors describing species has generally increased through time, but has largely remained stable since 1950 (Fig. 2C).

To visually represent the temporal distribution of indigenous and foreign authors, we have incorporated a colour-coded fraction in Fig. 2. This fraction helps distinguish the contributions made by each group over time, providing a clear visual representation of the disparities between endeavours of indigenous and foreign taxonomic. In addition, we have broken down the species description counts by author type for each year. This allows for a comprehensive comparison of the species descriptions attributed to indigenous versus foreign authors. Our analysis has revealed substantial disparities between these two groups, highlighting the contrasting levels of taxonomic effort and contributions. Figure 2A illustrates the stark difference in the number of species described by Nigerian researchers compared to foreign botanists. Similarly, Fig. 2C shows that only a fraction of the authors involved in describing Nigeria’s vascular plant species were of indigenous origin.

**Fig. 3.**
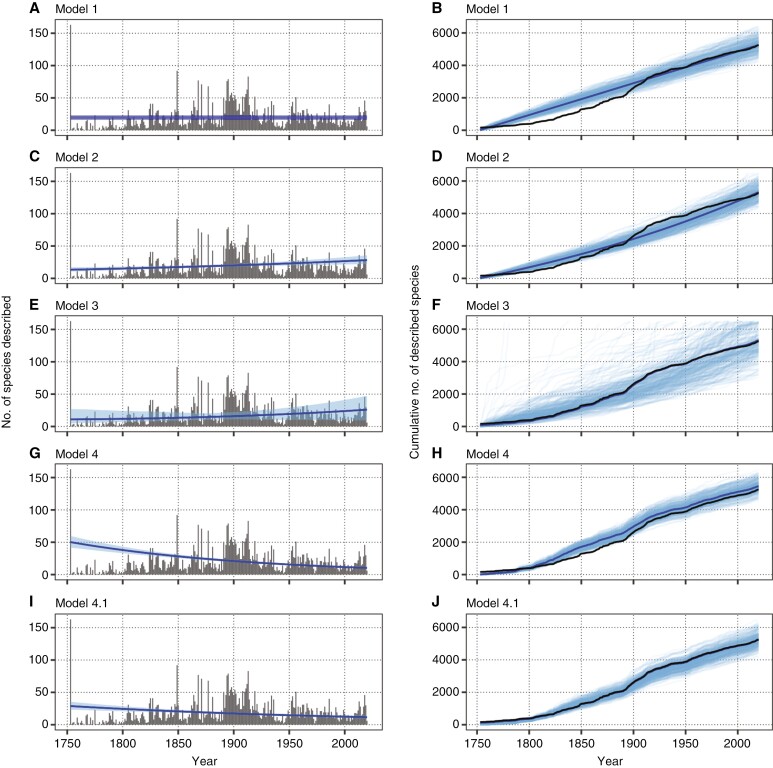
Model fits of species description trends in Nigerian vascular plants from 1753 to 2020. Left panels (A, C, E, G, I) show counts of species described per year as grey vertical bars, and the fitted long-term trend in those values is shown as the blue line (mean trend) and 90 % credible interval (light blue ribbon). Panels G and I show the expected trend in counts of species descriptions while accounting for number of authors per year. Right panels (B, D, F, H, J) show the cumulative counts of species described through time as the black line, with the mean model fit in dark blue and 250 samples from the model posterior in light blue.

An average of 19.5 species have been described per year (i.e. the intercept of model 1, Fig. 3A), which fits well with the observed rate of species description from ca. 1950 to today (Fig. 3B; Supplementary Data Fig. S1). Despite the initial pulse of description at the start of the study interval, the long-term trend in species description appears to credibly increase through time (i.e. the effect of *year* on description counts in model 2 is positive, see Fig. 3C). This log-linear increase in descriptions per year is better supported over the constant rate model 1 (Table 1), but it underpredicts the cumulative value through much of the 20th century (Fig. 3D). Incorporating a 1-year autoregressive term in model 3 improves model support over that for models 1 and 2 (Table 1), indicating that there are temporally local correlations of species counts. This model also removes any credible long-term trend in species description through time (Fig. 3E), suggesting that the positive long-term trend recovered by model 2 may spuriously emerge from these year-to-year correlations. The observed variance in year-to-year counts also introduces much broader uncertainty in the cumulative trajectories of species descriptions (Fig. 3F). Incorporating the number of authors as an offset in the rate of species description in model 4 has equivocal support with model 3 (Table 1). However, unlike models 2 and 3, after accounting for the increased probability of describing new species through an increase in active authors, the long-term trend in description counts is credibly negative (Fig. 3G). This model underpredicts counts in the initial part of the study interval, but overpredicts counts in the later part (Fig. 3H). Adding a 1-year autoregressive term in model 4.1 improves both model support and fit (Table 1; Fig. 3I, J), despite the increased parameterization. Here, the long-term trend in species description is credibly negative, as in model 4. Thus, we interpret the best-supported long-term trend in species description as declining through time.

### Forecasted species descriptions by 2070

**Fig. 4.**
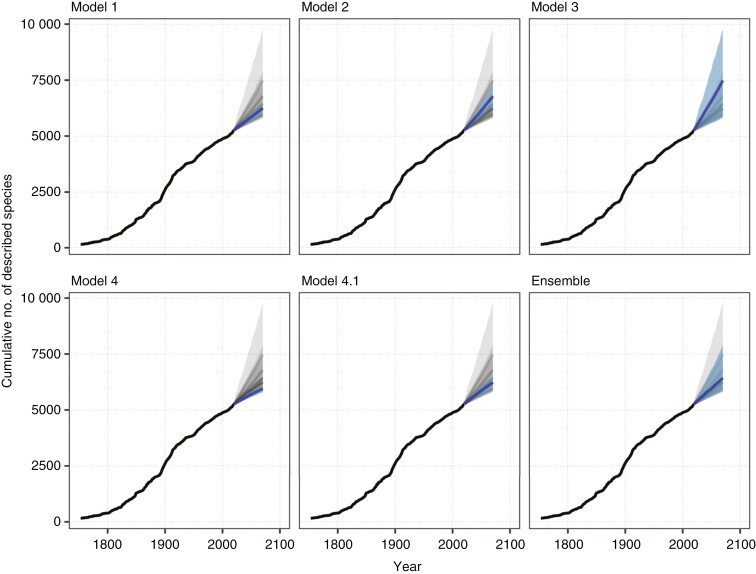
Forecasts of species descriptions by year 2070 for Nigerian vascular plants for each fitted model of species description. Individual panels show the mean forecast for the specified model from the year 2021 to 2070 as the dark blue line, with 90 % credible interval as the light blue ribbon. Dark grey lines and ribbons show forecasts for each model across all panels to facilitate comparison.

Given that the long-term trend in species description varies from positive (model 2) to constant (models 1 and 3) and negative (models 4 and 4.1), forecasts of species descriptions by the year 2070 also vary accordingly. If species descriptions continue at a constant rate of ~20 species per year, an additional 1004 [780–1247, 95 % credible interval] descriptions are expected by 2070 (Fig. 4: model 1). Assuming a log-linear trend in description through time results in a higher forecast (1539 [1120–2060]; Fig. 4: model 2), as does accounting for temporal autocorrelation (2239 [582–4537]; Fig. 4: model 3). After accounting for the number of authors per year, forecasts drop to 708 [544–881] (Fig. 4: model 4) and to 989 [721–1281] when also accounting for temporal autocorrelation (Fig. 4: model 4.1). Averaging predictions over the model posteriors, weighted by their expected log predictive density, estimates 1140 [593–2646] descriptions (Fig. 4: Ensemble).

## DISCUSSION

### Trends in species description rates for the Nigerian flora

This study has found that although the rate of species description has increased through time, the number of species described per author per year has declined since the first description in 1753. The study has found that the rate of description of species occurring in Nigeria has increased since 1753. This aligns with the general acceleration of scientific progress and discovery during the modern era. The initial description of 163 species in 1753 marked the start of the formal taxonomic classification system introduced by Carl Linnaeus (Bock, 2016). As scientists explored the world and had access to more specimens in the 19th century, the rate of species description grew substantially (Costello *et al*., 2013; Cheek *et al*., 2020). In the present study, this is reflected by a peak between 1890 and 1910 with over 20–30 new species that occur in Nigeria described per year.

The peak of species description coincided with the flourishing of natural history museums and advancements in microscopy, which undoubtedly aided the detection and documentation of new species (Wheeler, 2008). While the number of authors describing species increased significantly during this time, the rate of new species description has since stabilized, with most of the easily observable species on the planet already documented (Costello *et al*., 2013). The new species finds require more advanced techniques and exploration in remote regions (Mora *et al*., 2011). However, some estimates suggest that there are still 3–10 million eukaryotic species yet to be discovered (Mora *et al*., 2011; Christenhusz and Byng, 2016), leaving much work left for taxonomists and systematists to continue the 250-year history of species description that Linnaeus began (Bock, 2016; Sosef *et al*., 2017).

The decline in plant species description in Nigeria between 1900 and 1950 can be attributed to historical factors, including limited scientific exploration and prioritization during the colonial era, as well as knowledge and infrastructure gaps. The subsequent increase in species description from 1950 can be attributed to post-colonial developments, such as increased national focus, improved funding and international collaborations.

The general decline in species description until 2000 can be linked to factors such as a shift in scientific priorities, brain drain and capacity challenges. Nigeria experienced a brain drain of skilled taxonomists who are mostly Europeans and Indians holding academic positions in universities and other forestry and botanical organizations, with limited training opportunities for the indigenous botanists, which impacted the rate of species description. Other factors include limited resources and infrastructure, institutional weaknesses, political instability and economic challenges.

The finding that the number of species described by Nigerian researchers is relatively negligible compared to that by foreign botanists (Fig. 2A) raises important questions about the involvement and capacity of indigenous researchers in taxonomic endeavours. It suggests that there may be challenges or barriers faced by Nigerian researchers in effectively contributing to species description efforts. Similarly, the observation that only a fraction of the authors involved in describing Nigeria’s vascular plant species were of indigenous origin (Fig. 2C) indicates a potential imbalance in the representation and participation of Nigerian researchers in taxonomic studies. This finding calls for a closer examination of the factors contributing to this disparity and the need for greater inclusion and support for indigenous researchers.

These outcomes have several implications for biodiversity conservation. First, they highlight the urgency to address the taxonomic impediment and promote the development of local taxonomic expertise. Enhancing the capacity of Nigerian researchers and institutions in species description is crucial for accurately documenting and conserving the country’s rich biodiversity. Second, fostering collaborations between Nigerian and foreign researchers is essential for knowledge exchange, capacity building and enhancing research outcomes. Third, addressing the underlying factors contributing to the disparities, such as limited resources, infrastructure gaps and institutional weaknesses, is vital for creating an enabling environment for taxonomic research in Nigeria. This involves increased investment in taxonomic research, improvement of laboratory facilities and herbaria, and strengthening institutional support for botanical studies (Grieneisen *et al*., 2014). By acknowledging and addressing these disparities, Nigeria can foster a more inclusive and robust taxonomic community, contribute significantly to global biodiversity knowledge and work towards achieving the targets set by the GBF.

### Predictions of new vascular plant species descriptions in the next 50 years

This study forecasts the discovery of new vascular plant species through to 2070 under different predictions of continued species description. If the current trend of describing around 20 new vascular plant species per year continues for Nigeria, ~1004 [780–1247] new species will be described by 2070. However, models accounting for temporal autocorrelation and the number of authors predict greater uncertainty and lower numbers of new species descriptions by 2070, estimating around 593–2646 new species. An ensemble prediction that weights expected log predictive density estimates 1140 [593–2646] new vascular plant species will be described by 2070. These predictions point to the potential for a large number of new vascular plant species yet to be discovered, although at a slower rate of discovery than the current trend suggests. Continued exploration and documentation of plant biodiversity is needed to develop a more complete understanding of the diversity of vascular plants in Nigeria.

We cannot know the true number of undescribed species remaining in Nigeria with the data analysed here; we can only project estimates from short- and long-term trends. Nevertheless, it is important to consider how changes in the number of taxonomists may impact the goal of describing ~1000 additional species by 2070. Assuming the median number of active taxonomists per year from 2000 to the present day, which is 13, then meeting the ensemble forecasted value of 1140 species by 2070 would require ~1.75 descriptions per year per taxonomist. We may be nearer to a saturation of knowledge for Nigerian vascular plant diversity than we predict here, but the only way to test this is through continued sampling and taxonomy. Our estimates of missing diversity are likely to become more precise as more active taxonomists describe fewer species per year. Reductions in active taxonomists into the future will contribute further uncertainty to these predictions, and so continued support for the current and future generations of researchers and students is critical for making precise determinations of Nigerian vascular plant diversity, and biodiversity in general.

### Perspectives and implications for CBD’s post-2020 GBF

The results of our study on the discovery of new vascular plant species have important implications for Goal 1 and Goal 3 of the GBF, especially in the context of taxonomic impediments in African countries. First, our study suggests that there are potentially still a large number of new plant species yet to be discovered for Nigeria. This underlines the importance of continued exploration and documentation of plant biodiversity, as well as the need to address taxonomic impediments that may be hindering such efforts. Second, we stress the importance of mainstreaming biodiversity across government and society, as called for by Goal 1 of the GBF. In line with this, efforts to support and enable taxonomic research and assessment of biodiversity must be integrated into broader policy frameworks, so that they are given appropriate attention and resources. Third, as called for by Goal 3 of the GBF, biodiversity needs to be valued and conserved for the benefit of all people. Efforts to conserve and restore biodiversity must be undertaken with an understanding of the importance of plant diversity to ecosystem services and the well-being of local communities.

### A call to improve plant taxonomy research in Nigeria and other developing countries

Nigeria has a rich plant diversity, with many endemic species (Borokini, 2014; Bello *et al*., 2021). However, much of the knowledge about Nigerian plants comes from the work of non-Nigerian taxonomists. These early descriptions and reports provide a useful starting point, but do not fully reflect Nigeria’s plant diversity today. More research is needed by Nigerian botanists and taxonomists to provide a complete and up-to-date understanding of Nigeria’s flora. Several factors have contributed to the lack of recent taxonomic work on Nigerian plants. Limited funding for scientific research and higher education in Nigeria has made it difficult to pursue taxonomy and other plant sciences. Economic pressures have also drawn talented young scientists away from taxonomy towards more applied fields. Foreign researchers have played an important role in the past (Park, 2023), but Nigeria would benefit from building local expertise and institutional knowledge about its plant life. New taxonomic work should make use of established techniques such as DNA barcoding for aiding identification, and to clarify evolutionary relationships. Monographs should be produced for major plant groups such as grasses, orchids and legumes, which are especially diverse in Nigeria. Herbaria and research institutions should be strengthened and provided with modern equipment. With renewed focus and investment, Nigeria can build the expertise needed to comprehensively document its rich plant biodiversity and share this knowledge with the world. All these can be achieved by focusing on the following key points:

1. Investing in taxonomic research and supporting local taxonomists in tropical African countries is crucial to achieving the CBD’s GBF goal of putting biodiversity on a path to recovery by 2030 for the benefit of the planet and people. This aligns with Goal 1 of the GBF, which seeks to ‘address the direct and indirect drivers of biodiversity loss by mainstreaming biodiversity across government and society’.
2. Increasing collaboration and support for taxonomic research in tropical African countries, which are at particular risk of losing biodiversity due to the lack of comprehensive biodiversity assessments. This aligns with Goal 3 of the GBF, which seeks to ‘ensure that biodiversity is valued, conserved, restored and wisely used, maintaining ecosystem services, sustaining a healthy planet and delivering benefits essential for all people’.

Here, we provide some potential approaches to achieve these goals:

1. Funding opportunities: governments, foundations and other organizations can provide funding opportunities to support taxonomic research and training programmes in tropical African countries. These funds can be used to support research projects, provide scholarships for students, or provide training and mentorship opportunities for local taxonomists.
2. Collaboration and partnerships: international organizations and institutions can collaborate with local universities, research centres and government agencies to build capacity for taxonomic research in tropical African countries. These partnerships can provide access to resources, expertise and funding, while also facilitating the exchange of knowledge and skills.
3. Education and training: investing in education and training programmes can help to build the skills and knowledge of local taxonomists, as well as suppmme can include workshops, seminars and online courses that cover a range of topics related to taxonomy, including fieldwork techniques, specimen preparation and preservation, and data analysis.
4. Support for collections and infrastructure: taxonomic research relies on the availability of high-quality collections of specimens and other data. Investing in the development and maintenance of these collections, as well as the infrastructure needed to support them, can help to facilitate taxonomic research in tropical African countries.
5. Public engagement and awareness: building public awareness of the importance of taxonomic research can help to generate support for investment in this field. This can be achieved through public outreach programmes, such as workshops and seminars, as well as through media campaigns and social media outreach.
6. Recognition and support for local expertise: investing in taxonomic research and supporting local taxonomists in tropical African countries should also involve recognition and support for local expertise. This can include providing opportunities for local taxonomists to publish their work, attend conferences and workshops, and collaborate with researchers from other parts of the world.

## CONCLUSIONS

This study shows that the overall pattern of botanical species description in Nigeria is steadily increasing over time, with the number of described plant species rising rapidly from the early 19th century to the present day. However, this increase has been uneven across different taxonomic groups and regions within the country, with certain areas and groups being more thoroughly studied than others. Based on the historical trend of species description in Nigeria, it is estimated that ~905–1276 new vascular plant species will be described in the next 50 years. This estimate is subject to uncertainty due to variations in the rate of species description over time and the number of authors involved in the process. To achieve a comprehensive description of the unknown plant species in the country, a significant number of taxonomists will be needed for an extended period of time. The exact number of taxonomists required and the duration of the effort will depend on various factors, including the number of unknown species, the complexity of the taxonomic groups involved, and the availability of resources and infrastructure to support taxonomic research.

Overall, the study highlights the importance of continued taxonomic research in Nigeria and other countries with high levels of biodiversity. This research is essential for achieving the goals of the CBD’s post-2020 GBF, which seek to mainstream biodiversity across government and society and ensure that biodiversity is valued, conserved, restored, and wisely used for the benefit of all people and the planet. However, achieving these goals will require addressing the taxonomic impediments that currently hinder biodiversity assessment in many African countries, including Nigeria, by investing in taxonomic training, building taxonomic capacity, and supporting taxonomic research and infrastructure.

## SUPPLEMENTARY DATA

Supplementary data are available online at https://academic.oup.com/aob and consist of the following.

**Figure S1.** Posterior predictive checks for all models. **Table S1**. Checklist of vascular plants in Nigeria. **Table S2.** Priors for model 1 summary and performance. **Table S3.** Priors for model 2 summary and performance. **Table S4**. Priors for model 3 summary and performance. **Table S5**. Priors for model 4 summary and performance. **Table S6**. Priors for model 4.1 summary and performance.

mcad106_suppl_Supplementary_Materials
