# Supplementary material for: Trends in botanical exploration in Nigeria forecast over 1000 yet undescribed vascular plant species
Source: Ann Bot. 2024 May 10;133(5-6):789–800. doi: 10.1093/aob/mcad106 (PMC11082469; doi:10.1093/aob/mcad106)
Supplement: mcad106_suppl_Supplementary_Materials [file mcad106_suppl_supplementary_materials.zip › aob-23133-s03.docx]

Supplementary figure caption

[**Figure S1.**](#bookmark=id.35nkun2) Posterior predictive checks for all models. Observed density of species descriptions per year show in dark blue (*y*), with 100 draws from each posterior distribution shown in light blue (*y_rep_*).
