# Supplementary material for: Trends in botanical exploration in Nigeria forecast over 1000 yet undescribed vascular plant species
Source: Ann Bot. 2024 May 10;133(5-6):789–800. doi: 10.1093/aob/mcad106 (PMC11082469; doi:10.1093/aob/mcad106)
Supplement: mcad106_suppl_Supplementary_Materials [file mcad106_suppl_supplementary_materials.zip › aob-23133-s04.docx]

# **SUPPLEMENTAL MATERIAL FOR:**

**Trends in botanical exploration in Nigeria forecast over 100 yet undescribed vascular plant species**

Abubakar Bello, Stewart M. Edie, Kowiyou Yessoufou, Alexandra Nora Muellner-Riehl

**Supplemental Analyses**

***Models of species description.***

Details on model fits provided in the tables below.

[Table S2.](#stabl_smym1) Priors for [model 1](#mod_1) summary and performance.

| **PRIORS**  **prior class** student_t(3, 2.7, 2.5) Interceptgamma(0.01, 0.01) shape |
| --- |
| **MODEL SUMMARY AND PERFORMANCE** Family: negbinomial Links: mu = log; shape = identityFormula: count ~ 1 Data: mDATA (Number of observations: 268) Draws: 4 chains, each with iter = 7500; warmup = 1000; thin = 10; total post-warmup draws = 2600Population-Level Effects: Estimate Est.Error l-95% CI u-95% CI Rhat Bulk_ESS Tail_ESSIntercept 2.9741 0.0557 2.8656 3.0851 0.9997 2790 2557Family Specific Parameters: Estimate Est.Error l-95% CI u-95% CI Rhat Bulk_ESS Tail_ESSshape 1.2978 0.1140 1.0873 1.5358 1.0000 2362 2553Draws were sampled using sampling(NUTS). For each parameter, Bulk_ESSand Tail_ESS are effective sample size measures, and Rhat is the potentialscale reduction factor on split chains (at convergence, Rhat = 1). |

[Table S3.](#stabl_smym2) Priors for [model 2](#mod_2) summary and performance.

| **PRIORS** **prior class coef** (flat) b (flat) b yearstudent_t(3, 2.7, 2.5) Intercept gamma(0.01, 0.01) shape |
| --- |
| **MODEL SUMMARY AND PERFORMANCE** Family: negbinomial Links: mu = log; shape = identityFormula: count ~ year Data: mDATA (Number of observations: 268) Draws: 4 chains, each with iter = 7500; warmup = 1000; thin = 10; total post-warmup draws = 2600Population-Level Effects: Estimate Est.Error l-95% CI u-95% CI Rhat Bulk_ESS Tail_ESSIntercept -2.3302 1.5264 -5.2780 0.6997 1.0007 2643 2385year 0.0028 0.0008 0.0012 0.0044 1.0007 2648 2459Family Specific Parameters: Estimate Est.Error l-95% CI u-95% CI Rhat Bulk_ESS Tail_ESSshape 1.3533 0.1241 1.1305 1.6163 1.0014 2655 2534Draws were sampled using sampling(NUTS). For each parameter, Bulk_ESSand Tail_ESS are effective sample size measures, and Rhat is the potentialscale reduction factor on split chains (at convergence, Rhat = 1). |

[Table S4.](#stabl_smym3) Priors for [model 3](#mod_3) summary and performance.

| **PRIORS** **prior class coef** (flat) ar (flat) b (flat) b year student_t(3, 2.7, 2.5) Intercept student_t(3, 0, 2.5) sderr gamma(0.01, 0.01) shape |
| --- |
| Family: negbinomial Links: mu = log; shape = identityFormula: count ~ year + ar(time = year, p = 1, cov = T) Data: mDATA (Number of observations: 268) Draws: 4 chains, each with iter = 15000; warmup = 2000; thin = 10; total post-warmup draws = 5200Correlation Structures: Estimate Est.Error l-95% CI u-95% CI Rhat Bulk_ESS Tail_ESSar[1] 0.8032 0.0831 0.6218 0.9558 1.0148 391 149sderr 0.4678 0.0918 0.3158 0.6726 1.0022 1679 1879Population-Level Effects: Estimate Est.Error l-95% CI u-95% CI Rhat Bulk_ESS Tail_ESSIntercept -3.9745 5.9816 -12.2001 9.1696 1.0113 503 150year 0.0035 0.0032 -0.0033 0.0079 1.0123 504 150Family Specific Parameters: Estimate Est.Error l-95% CI u-95% CI Rhat Bulk_ESS Tail_ESSshape 6.3124 11.0587 2.9734 18.0178 1.0026 1655 1741Draws were sampled using sampling(NUTS). For each parameter, Bulk_ESSand Tail_ESS are effective sample size measures, and Rhat is the potentialscale reduction factor on split chains (at convergence, Rhat = 1). |

[Table S5.](#stabl_smy4) Priors for [model 4](#mod_4) summary and performance.

| **PRIORS** **prior class coef** (flat) b (flat) b year student_t(3, 0.921652924780976, 2.5) Intercept gamma(0.01, 0.01) shape |
| --- |
| **MODEL SUMMARY AND PERFORMANCE** Family: negbinomial Links: mu = log; shape = identityFormula: count ~ year + offset(log(numauthor)) Data: mDATA (Number of observations: 268) Draws: 4 chains, each with iter = 15000; warmup = 2000; thin = 10; total post-warmup draws = 5200Population-Level Effects: Estimate Est.Error l-95% CI u-95% CI Rhat Bulk_ESS Tail_ESSIntercept 12.1621 1.0800 10.0692 14.2828 0.9997 5357 5100year -0.0059 0.0006 -0.0070 -0.0048 0.9998 5372 5020Family Specific Parameters: Estimate Est.Error l-95% CI u-95% CI Rhat Bulk_ESS Tail_ESSshape 2.2669 0.2209 1.8540 2.7231 1.0003 5272 5013Draws were sampled using sampling(NUTS). For each parameter, Bulk_ESSand Tail_ESS are effective sample size measures, and Rhat is the potentialscale reduction factor on split chains (at convergence, Rhat = 1). |

###

[Table S6.](#stabl_smy4.1) Priors for [model 4](#mod_4).1 summary and performance.

| **PRIORS**  **prior class coef** (flat) ar (flat) b (flat) b year student_t(3, 0.921652924780976, 2.5) Intercept student_t(3, 0, 2.5) sderr gamma(0.01, 0.01) shape |
| --- |
| **MODEL SUMMARY AND PERFORMANCE** Family: negbinomial Links: mu = log; shape = identityFormula: count ~ year + offset(log(numauthor)) + ar(time = year, p = 1, cov = T) Data: mDATA (Number of observations: 268) Draws: 4 chains, each with iter = 15000; warmup = 2000; thin = 10; total post-warmup draws = 5200Correlation Structures: Estimate Est.Error l-95% CI u-95% CI Rhat Bulk_ESS Tail_ESSar[1] 0.2621 0.1135 0.0320 0.4775 1.0008 4257 4570sderr 0.5293 0.0367 0.4541 0.6004 1.0007 3851 4171Population-Level Effects: Estimate Est.Error l-95% CI u-95% CI Rhat Bulk_ESS Tail_ESSIntercept 7.1839 1.2956 4.6868 9.8473 1.0005 4363 4614year -0.0034 0.0007 -0.0048 -0.0021 1.0006 4385 4300Family Specific Parameters: Estimate Est.Error l-95% CI u-95% CI Rhat Bulk_ESS Tail_ESSshape 67.7626 65.4860 12.8263 248.2891 0.9999 3281 4104Draws were sampled using sampling(NUTS). For each parameter, Bulk_ESSand Tail_ESS are effective sample size measures, and Rhat is the potentialscale reduction factor on split chains (at convergence, Rhat = 1). |

### 
